# Sirtuin-dependent reversible lysine acetylation of the *o*-succinylbenzoyl-coenzyme A synthetase of *Bacillus subtilis*

**DOI:** 10.1128/spectrum.02011-24

**Published:** 2024-10-18

**Authors:** Rachel M. Burckhardt, Jorge C. Escalante-Semerena

**Affiliations:** 1 Department of Microbiology, University of Georgia, Athens, Georgia, USA; University of Minnesota, St. Paul, Minnesota, USA

**Keywords:** metabolic stress, Gcn5-related acetyltransferases, menaquinone biosynthesis, sirtuin deacylase, *Bacillus subtilis *metabolism, proton motive force control, OSB-CoA synthetase

## Abstract

Reversible lysine acylation (RLA) is a conserved posttranslational modification that cells of all domains of life use to regulate the biological function of proteins, some of which have enzymatic activity. Many AMP-forming organic acid:CoA ligases are regulated *via* acylation in prokaryotes and eukaryotes. Here, we report the acetylation of the *o*-succinylbenzoyl-CoA synthetase (EC 6.2.1.26) of *Bacillus subtilis* (*Bs*MenE) by the GCN5-related acetyltransferase (GNAT) AcuA enzyme of this bacterium. *Bs*MenE is part of the metabolic pathway that assembles menaquinone (MK), an essential component of the electron transport chain in *B. subtilis*. We demonstrate that the active-site lysine 471 (K471) of *Bs*MenE is acetylated specifically by *Bs*AcuA, and that acetylated *Bs*MenE (*Bs*MenE^Ac^) is deacetylated by the NAD^+^-dependent sirtuin (*Bs*SrtN) of this bacterium. The *in vivo* analyses performed in this study were done in an *Escherichia coli* Δ*menE* strain because the enzymatic activity of MenE is essential in *B. subtilis*, but not in *E. coli*. The use of a heterologous system allowed us to assess the effect of acetylation on *Bs*MenE function under MK-dependent growth conditions. Based on our *in vivo* data, we suggest that regulation of *Bs*MenE by RLA reduces MK production, negatively affecting the growth rate and yield of the culture.

**IMPORTANCE:**

Reversible lysine acylation (RLA) is a posttranslational modification used by all cells to rapidly control the biological function of proteins. Herein, we identify an acetyltransferase and deacetylase in the soil bacterium *Bacillus subtilis* that can modify/demodify an enzyme required for the synthesis of menaquinone (MK), an essential electron carrier involved in respiration in cells of all domains of life. Based on our data, we suggest that under some as-yet-undefined physiological conditions, *B. subtilis* modulates MK biosynthesis, which changes the flux of electrons through the electron transport chain of this bacterium. To our knowledge, this is the first example of control of respiration by RLA.

## INTRODUCTION

Posttranslational modifications (PTMs) allow cells to respond quickly to stimuli and cope with environmental and cellular stress (1, 2). Acetylation of the *Nε* group of the side chain of lysine is a common PTM found in cells from all domains of life (3) and has been shown to regulate cellular processes, such as transcription (4), small-molecule detoxification (5), cell structure (6, 7), nucleoid compaction (8), metabolism (9–11), translation (12, 13), among others.

Some types of PTMs are reversible, allowing for up- or downregulation of the biological function of proteins (1). If the acetyl moiety of acetyl-Lys (AcK) can be removed, this type of regulation referred to as reversible lysine acetylation (RLA).

Reactions 1 and 2 below show how proteins are reversibly acetylated to control their biological function. Below, we show that, in some cases, reaction 2 is catalyzed by NAD^+^-dependent deacetylases (a.k.a. sirtuins).

Protein + Ac-CoA + *N*-acetyltransferase → Proten^Ac^ +CoA ………………………… (Rxn 1)

Protein^Ac^ + NAD^+^ + sirtuin deacylase → Protein + *O-*AADPR* +Nm ……………….. (Rxn 2)

*, *O-*AADPR = *O-*acetyl-ADP-ribose; Nm = nicotinamide

Acetylation has been shown to modify protein function by altering stability, structure, or activity (14). In most cases described to date, deacetylation reactivates the biological function of the target protein (9, 15–17).

From the standpoint of metabolism, the paradigm of RLA in all forms of life is the regulation of AMP-forming acetyl-CoA synthetase (Acs) enzymes (IPR000873) (9). This family of enzymes activates an organic acid to its corresponding CoA thioester at the expense of ATP *via* an acyl-AMP intermediate (18). GCN5-related acetyltransferase (GNAT) acetyltransferases modify the epsilon amino group of lysyl residues critical for the adenylylation reaction, *i.e.*, the first half of the reaction (18, 19). GNAT-dependent RLA regulation of AMP-forming CoA synthetases has been shown in several prokaryotes, namely *Salmonella enterica* subsp. *enterica* sv. Typhimurium str. LT2 (hereafter *S*. Typhimurium), *Rhodopseudomonas palustris*, *Streptomyces lividans*, *Saccharopolyspora erythraea*, *Mycobacterium tuberculosis*, *Staphylococcus aureus*, *Campylobacter jejuni*, and others (10, 20–25). In all the above-mentioned examples the deacetylase involved belongs to the class III deacylases or sirtuins, which require NAD^+^ for function (26). Thus, it appears that RLA is a conserved mechanism to regulate AMP-forming CoA synthetases. Unlike all of the abovementioned bacteria, *Bacillus subtilis Bs*AcsA^Ac^ is reactivated by an NAD^+^-independent histone deacylase, not rather than a NAD^+^-dependent sirtuin (27).

**Fig 1:**
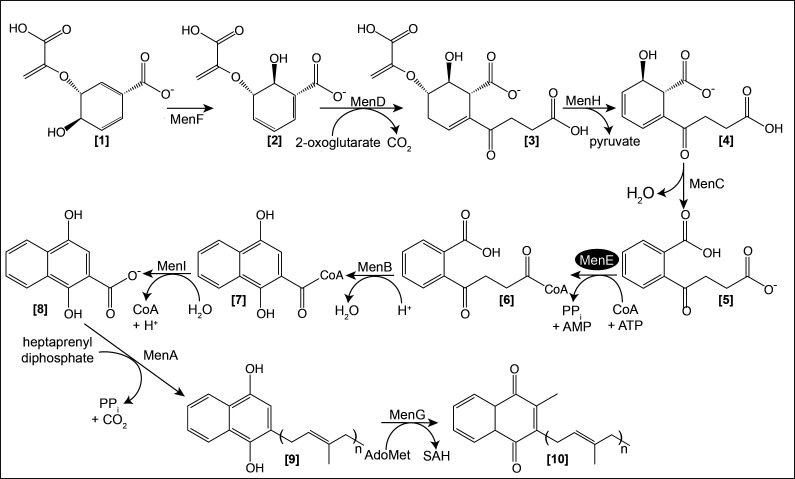
Menaquinone-7 biosynthesis in *Bacillus subtilis*. This pathway has been reproduced from BsubCyc (https://biocyc.org/BSUB/NEW-IMAGE?type=PATHWAY&object=PWY-5840&detail-level=4). Pathway intermediates are identified by boldface type bracketed numbers: [1], chorismate; [2], isochorismate; [3], 2-succinyl-5-enolpyruvoyl-6-hydroxy-3-cyclohexene-1-carboxylate (SEPHCHC); [4], 1*R*, 6*R*-6-hydroxy-2-succinylhexa-2,4-diene-1-carboxylate (SHCHC); [5], *o-*succinylbenzoate (OSB), [6], *o-*succinylbenzoyl-CoA (OSB-CoA); [7], 1,4-dihydroxy-2-naphthoyl-CoA DHNA-CoA); [8], 1,4-dihydroxy-2-naphthanoate (DHNA); [9], demethylmenaquinol-7 (*n* = 7); [10], menaquinone (MK, *n* = 7) a.k.a MK-7). The enzymes involved are MenF (isochorismate synthase, EC 5.4.4.2), MenD (2-ketoglutarate decarboxylase, SEPHCHC synthase, EC 2.2.1.9), MenH (SHCHC synthase, EC 4.2.99.20), MenC (OSB synthase, EC 4.2.1.113), MenE (OSB-CoA ligase, EC 6.2.1.26), MenB (DHNA-CoA synthase, EC 4.1.3.36), MenI (DHNA-CoA hydrolase, EC:3.1.2.28), MenA (DHNA:heptaprenyltransferase), and MenG (MK methyltransferase, EC 2.1.1.163).

Many cellular processes involve the activity of AMP-forming acyl-CoA synthetases. An example relevant to the work reported here is found in the menaquinone (MK) biosynthetic pathway in *B. subtilis*. A critical step in this pathway is the activation of *o*-succinylbenzoate (OSB) into *o*-succinylbenzoyl-CoA (OSB-CoA) by MenE (Fig. 1, hereafter *Bs*MenE). Four additional steps convert OSB-CoA to menaquinone (MK) (Fig. 1). MK is a lipid-soluble redox component of the electron transport chain that mediates the transfer of electrons between dehydrogenases and cytochromes (28). MK is the sole quinone in Gram-positive bacteria, while facultative Gram-negative bacteria use MK when growing anoxically, and ubiquinone (UBK) during growth in the presence of molecular oxygen (28).

Here, we show that the *o*-succinylbenzoyl-CoA synthetase activity of *Bs*MenE is reversibly acetylated by the type IV GCN-5-related *Bs*AcuA acetyltransferase (GNAT, IPR024035) and the *Bs*SrtN deacylase enzymes of this bacterium. Our data raise the possibility that under some as-yet-undefined conditions, *B. subtilis* uses RLA to modulate the activity of *Bs*MenE, and thus MK production. This regulation may be a novel means for controlling the magnitude of the proton motive force (PMF) in this and possibly other prokaryotes.

## RESULTS

### The acetylation motif of AMP-forming acyl-CoA synthetases

**Fig 2:**
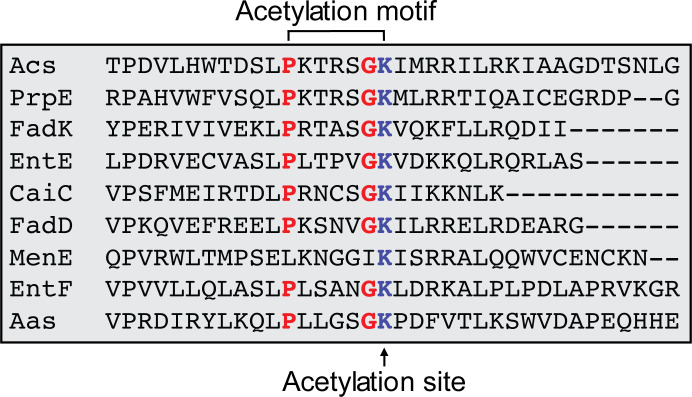
Alignment of the putative acetylation motif present in the C termini of AMP-forming acyl-CoA synthetases from *E. coli*. The PX_4_GK constitutes the so-called acetylation motif in the protein family IPR000873. Acs, acetyl-CoA synthetase (EC 6.2.1.1); PrpE, propionyl-CoA synthetase (EC 6.2.1.17); FadK, fatty acyl-CoA synthetase; EntE, enterobactin synthase component B (EC 6.3.2.14); CaiC, crotonobetaine/carnitinoyl-CoA synthetase (EC 6.2.1.48); FadD, long-chain fatty acid-CoA ligase (EC 6.2.1.3); MenE, *o-*succinylbenzoyl-CoA synthetase (EC 6.2.1.26); EntF, enterobactin synthase, component F (EC 6.3.2.14); Aas, 2-acyl-glycerolphophoethanolamine acyltransferase and acyl-acyl carrier protein synthetase.

The initial approach to defining an acetylation motif in proteins consisted of alignments of the C-termini of AMP-forming acyl-CoA synthetases present in the enterobacterium *Escherichia coli* K12 MG1655 (9); an example of such an alignment is shown in Fig. 2. The analysis of this alignment revealed that the MenE enzyme (OSB-CoA ligase, EC 6.2.1.26) of this bacterium had a Pro-to-Leu substitution at position −6 relative to the putative acetylation site. This and other differences within the MenE acetylation motif raised the question of whether the activity of *Ec*MenE was controlled by RLA.

**Fig 3:**
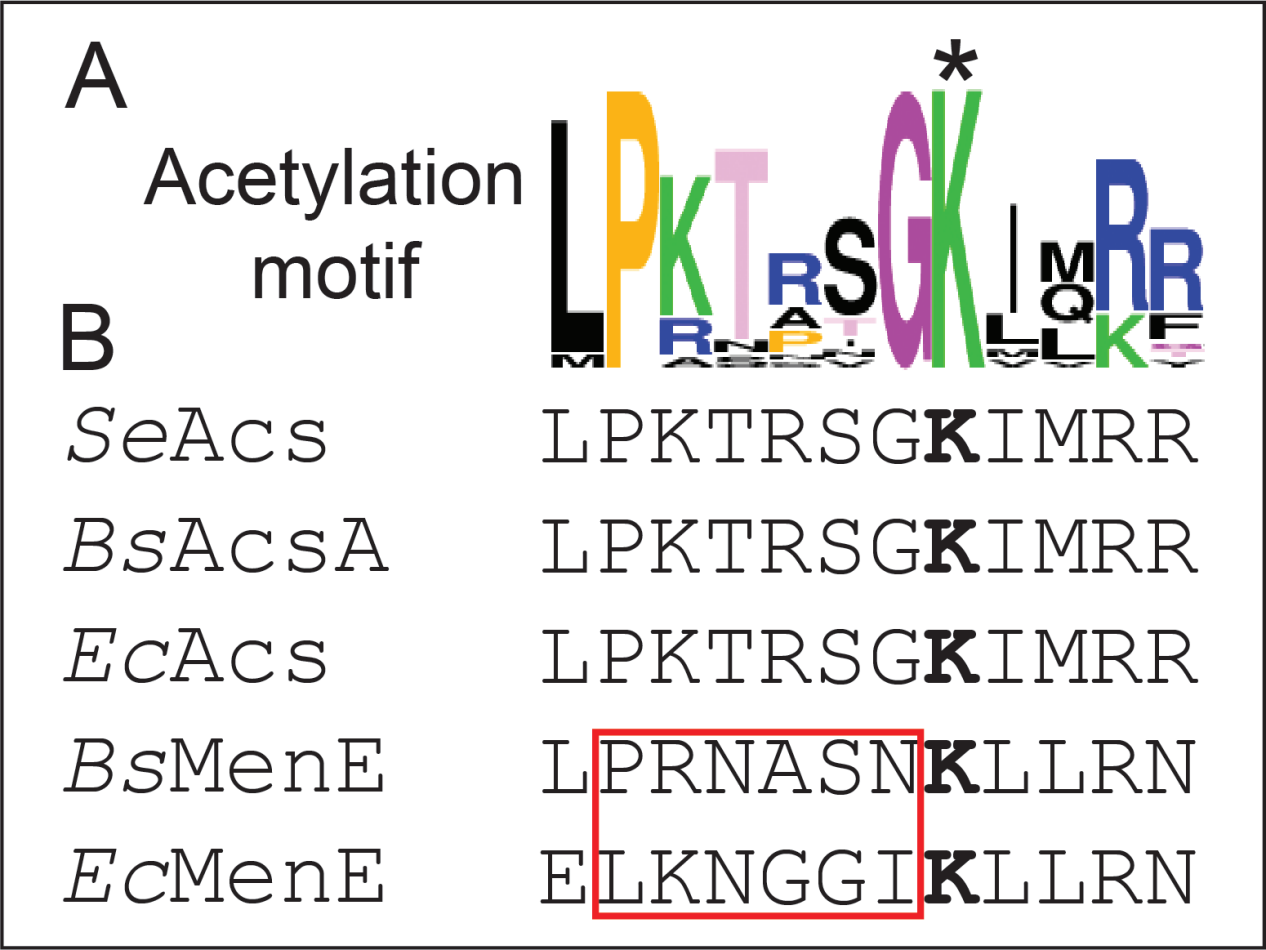
Comparison of the acetylation motifs of *Bs*MenE and *E. coli* to those of RLA-controlled AMP-forming CoA ligases. Primary sequence of the acetylation motif *bona fide* RLA-controlled acetyl-CoA synthetases from *S*. Typhimurium (*Se*Acs), *B. subtilis* (*Bs*AcsA), and *E. coli* (*Ec*Acs). *, putative acetylation site in *Bs*MenE and *Ec*MenE. The weblogo shown was generated as described under Materials and Methods.

In contrast to *Ec*MenE, *Bs*MenE has a Pro residue at position −6 (Fig. 3B); hence, we speculated that *Bs*MenE might be regulated by RLA, and that such regulation could impact cell growth. As a point of reference, Fig. 3A includes acetylation motifs of *bona fide* RLA-controlled acetyl-CoA synthetases from *S*. Typhimurium Acs (*Se*Acs), *E. coli* Acs (*Ec*Acs), and *B. subtilis* Acs (*Bs*AcsA).

### *Bs*AcuA acetylates *Bs*MenE once on residue K471

**Fig 4:**
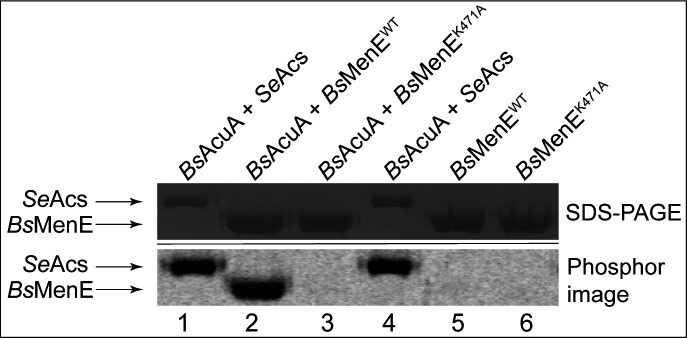
*Bs*AcuA acetylates residue K471 of *Bs*MenE. As a positive control, we incubated *Bs*AcuA with [1-^14^C]Ac-CoA and *Se*Acs (lanes 1 and 4). Results of experiments with *Bs*MenE^WT^ and *Bs*MenE^K471A^ are shown in lanes 2 and 3, respectively. *Bs*MenE^WT^ (lane 5) and *Bs*MenE^K471A^ (lane 6) were also incubated with [1-^14^C]Ac-CoA in the absence of *Bs*AcuA to demonstrate dependence on the activity of the acetyltransferase. Proteins were resolved by SDS-PAGE and stained with Coomassie Blue G-250. Protein acetylation was visualized by phosphor imaging as described under Materials and Methods.

The conserved lysine within the acetylation motif of *Bs*MenE corresponds to residue 471 (Fig. 3). Given that many AMP-forming acyl-CoA synthetases are acylated at this position, we suspected that K471 of *Bs*MenE was acetylated, but did not know which acyltransferase would modify it. We tested *in vitro* whether the lysine acetyltransferase *Bs*AcuA could modify *Bs*MenE, since *Bs*AcuA was known to acetylate *Bs*AcsA (29). Details of the purification protocol for the isolation of proteins used in our assays are described under Materials and Methods. In one set of assays, purified proteins were incubated with [1-^14^C]Ac-CoA, and as shown in Fig. 4, *Bs*AcuA acetylated *Bs*MenE (Fig. 4A, lanes 2 vs 5). A control reaction mixture contained *Se*Acs, which has been shown to be a substrate for *Bs*AcuA (19). These results mimicked those reported for other AMP-forming acyl-CoA synthetases (9, 21, 23, 24, 30).

**Fig 5:**
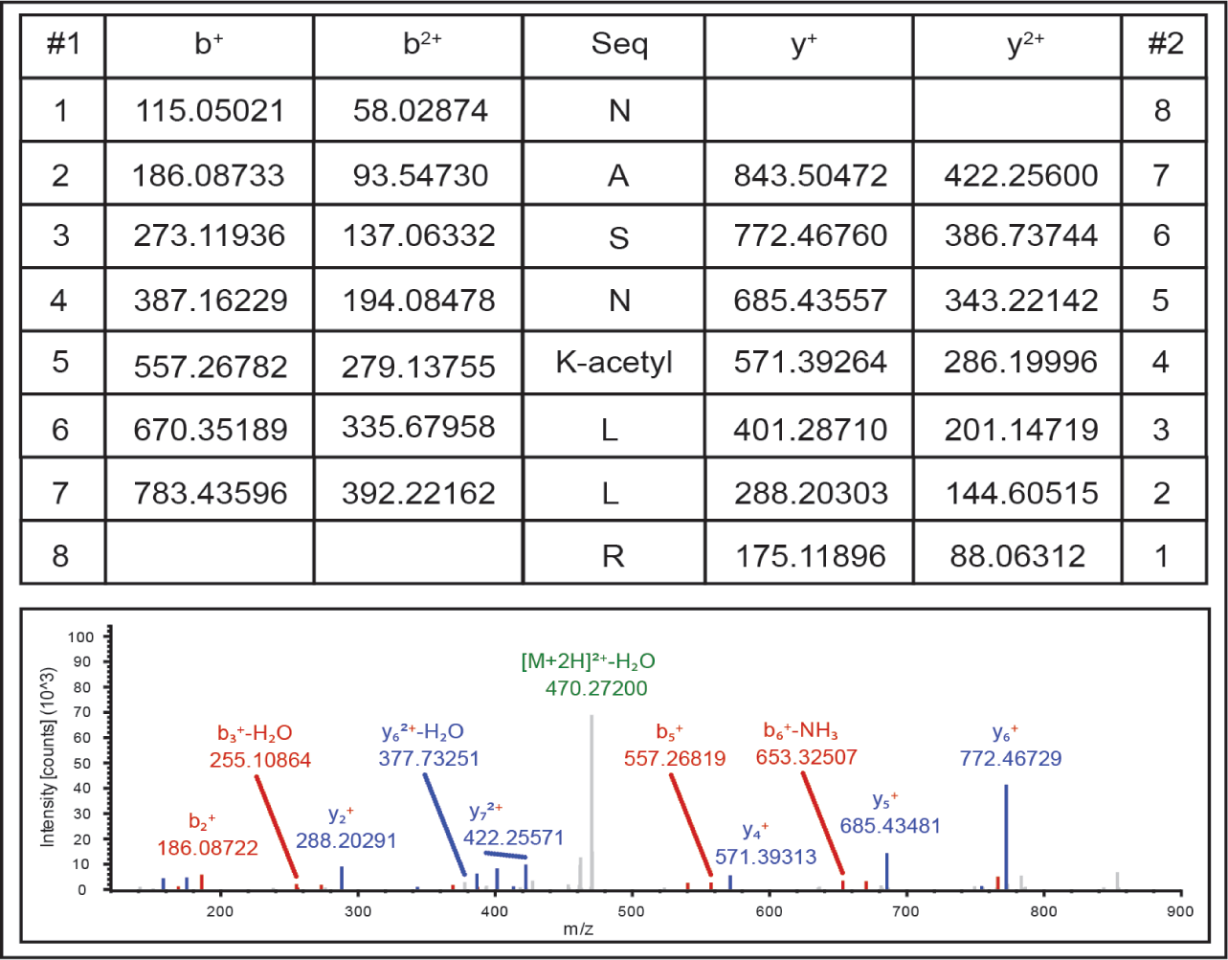
Mass spectrometry peptide finger printing analysis of acetylated *Bs*MenE. The conditions for data acquisition and analysis are described under Materials and Methods. The data support the conclusion that *Bs*MenE residue K471 is acetylated by *Bs*AcuA at the expense of Ac-CoA.

A separate set of assays was performed with non-radiolabeled Ac-CoA for the purpose of identifying the number and location(s) of acetyl moieties transferred by *Bs*AcuA onto *Bs*MenE. For this purpose, we isolated the *Bs*MenE from reaction mixtures after incubation with *Bs*AcuA plus Ac-CoA, and peptide finger printing mass spectrometry was performed on *Bs*MenE. As shown in Fig. 5, LC/MS/MS analysis unequivocally showed that K471 was acetylated by *Bs*AcuA; no other acetylation site was detected by this analysis. The MS data were consistent with the inability of *Bs*AcuA to modify *Bs*MenE^K471A^ (Fig. 4, lane 3).

### *Bs*MenE acetylation by *Bs*AcuA is reversed by the *B. subtilis* NAD^+^-dependent sirtuin deacetylase (*Bs*SrtN)

**Fig 6:**
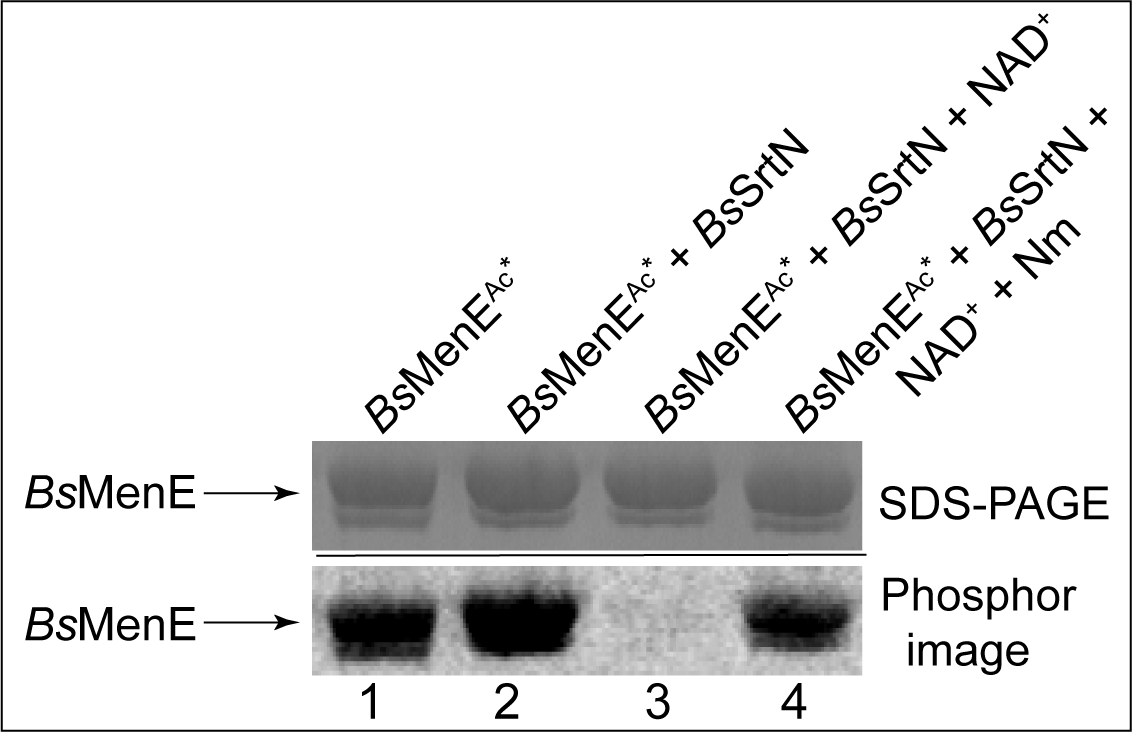
*Bs*SrtN deacetylates *Bs*MenE^Ac^ at the expense of NAD^+^. *Bs*MenE was acetylated by *Bs*AcuA using [1-^14^C]Ac-CoA as substrate. After removal of excess [1-^14^C]Ac-CoA (see Materials and Methods), the sample was used to generate four reaction mixtures. Nothing was added to the first reaction mixture (lane 1); to the second reaction mixture, we added *Bs*SrtN (lane 2); to the third reaction mixture, we added *Bs*SrtN and NAD^+^ (1 mM) (lane 3), and to the fourth reaction mixture, we added *Bs*SrtN, NAD^+^ (1 mM) and nicotinamide (Nm; 1 mM) (lane 4). The upper panel shows the SDS-PAGE gel stained with Coomassie Blue G-250 containing *Bs*MenE^Ac*^; the lower panel shows the phosphor image of the gel. The asterisk indicates that the acetyl moiety modifying *Bs*MenE was radiolabeled.

The above results prompted us to ask whether *Bs*MenE^Ac^ could be deacetylated by the NAD^+^-dependent sirtuin deacetylase (*Bs*SrtN) of this bacterium (15). To test this possibility, *Bs*MenE was acetylated by *Bs*AcuA using [1-^14^C]Ac-CoA. The resulting radiolabeled *Bs*MenE^Ac*^ (the asterisks indicate that the acetyl moiety modifying *B*sMenE was radiolabeled) was incubated in the absence or presence of *Bs*SrtN (Fig. 6, lanes 1 and 2), in the presence of *Bs*SrtN ± NAD^+^ (Fig. 6, lanes 2 and 3), and in the presence of *Bs*SrtN + NAD^+^ ± nicotinamide (Nm; Fig. 6, lanes 3 and 4). As shown in Fig. 6, lanes 2 and 3, incubation of *Bs*MenE^Ac^ with *Bs*SrtN and NAD^+^ resulted in a loss of signal associated with radiolabeled *Bs*MenE^Ac*^ (Fig. 6, lane 1), indicating that *Bs*SrtN deacetylated *Bs*MenE^Ac*^ at the expense of NAD^+^. We also monitored the deacetylation of *Bs*MenE^Ac*^ in a reaction mixture that included nicotinamide (Nm), a known inhibitor of sirtuins. Based on the data shown Fig. 6 (lane 4), we concluded that *Bs*SrtN was responsible for the deacetylation of *Bs*MenE^Ac*^. Collectively, our data indicated that *Bs*MenE acetylation was reversible.

### *Ec*MenE is not acetylated by GNATs from different bacteria

**Fig 7:**
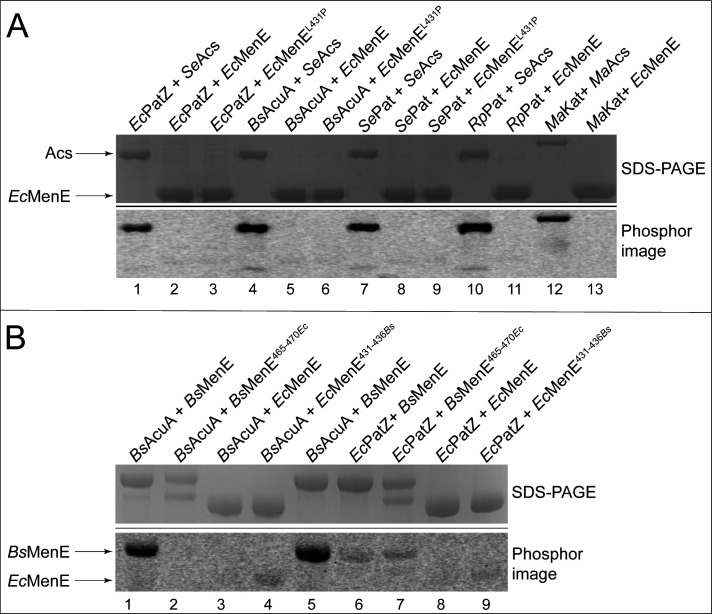
*Ec*MenE is not acetylated. (**A**) Various acetyltransferases were incubated with [1-^14^C]Ac-CoA and *Se*Acs, *Ma*Acs or *Ec*MenE. Purified acetyltransferases from *E. coli* (*Ec*PatZ, lanes 1–3), *B. subtilis* (*Bs*AcuA; lanes 4–6), *S*. Typhimurium (*Se*Pat, lanes 7–9), *Rhodopseudomonas palustris* (*Rp*Pat; lanes 10–11), and *Micromonospora aurantiaca* (*Ma*Kat, lanes 12–13) were tested. Proteins were resolved on an SDS-PAGE. Shown is the Coomassie-stained gel (top) and the phosphor image (bottom). (**B**) Acetyltransferases *Bs*AcuA (lanes 1–4) and *Ec*PatZ (lanes 6–9) were incubated with [1-^14^C]Ac-CoA and chimeras *Bs*MenE^465-470*Ec*^ and *Ec*MenE^431-436*Bs*^. Proteins were resolved on a SDS-PAGE. Shown are the Coomassie-stained gel (top) and the phosphor image (bottom).

Since *Bs*MenE was acetylated *in vitro*, we wanted to determine whether *Ec*MenE could also be acetylated by its cognate GNAT PatZ (a.k.a. YfiQ, Pka). *Ec*PatZ acetylates *Ec*Acs on residue K609, just as *Bs*AcuA acetylates *Bs*AcsA at the equivalent location (27, 31). *Ec*PatZ and other AMP-forming, acyl-CoA synthetase-acetylating GNATs from a variety of bacteria were also tested, but none acetylated *Ec*MenE (Fig. 7A, lanes 2, 5, 8, 11, and 13). All GNATs were active since all acetylated the acetyl-CoA synthetase *Se*Acs from *S*. Typhimurium (Fig. 7A, lanes 1, 4, 7, and 10), or from *Micromonospora aurantiaca* (*Ma*Acs, Fig. 7A, lane 12). Taken together, these data implied that *Ec*MenE was not recognized by the CoA synthetase-acetylating GNATs we tested. Thus, we hypothesized that *Ec*MenE might not be under RLA control in *E. coli*.

To acetylate *Ec*MenE, the leucine located six residues upstream (position −6) from the putative acetylation site was changed to proline to make the putative acetylation motif of *Ec*MenE more like consensus (Fig. 3). However, the *Ec*MenE^L431P^ variant (encoded by plasmid pEcMENE4) was not acetylated by either the acetyltransferase from *E. coli* (Fig. 7A, lane 3), *B. subtilis* (Fig. 7A, lane 6), or *S*. Typhimurium (Fig. 7A, lane 9). Since multiple residues around the acetylated lysine have been shown to be important for recognition by a GNAT (32), we changed all six residues before the putative acetylated lysine residue of *Ec*MenE to the residues found in those positions of *Bs*MenE (Fig. 3B, red box). The resulting chimera protein *Ec*MenE^431-436*Bs*^ (encoded by pEcMENE5) was purified and tested for acetylation by *Bs*AcuA or *Ec*PatZ. The sequence of the acetylation motif of *Bs*MenE did allow *Bs*AcuA and *Ec*PatZ to recognize *Ec*MenE^431-436*Bs*^ chimera to be acetylated (Fig. 7B, lanes 4 vs 5, lanes 5 vs 9, respectively), albeit to a lesser extent than *Bs*MenE, suggesting that changes inside and outside the acetylation motif of *Ec*MenE would be needed to improve recognition of *Ec*MenE by *Ec*PatZ. We also performed the reciprocal experiment with *Bs*MenE, changing the six residues before the acetylated lysine to those residues found in *Ec*MenE. The resulting chimera (*Bs*MenE^E465-470*Ec*^) was not acetylated by neither *Bs*AcuA nor *Ec*PatZ (Fig. 7B, lanes 1 vs 2 and 7). The identity of the protein with the very faint signal in Fig. 7B, lane 7 was not identified. These data showed that the six residues before K471 of *Bs*MenE were necessary but not sufficient for acetylation by *Bs*AcuA. More work is needed to understand the specificity of *Bs*AcuA for its substrates.

### *Bs*MenE from *B. subtilis* 168 has been found to be acetylated *in vivo*

We found support for our findings by searching the Compendium of Protein Lysine Modifications database (http://cplm.biocuckoo.cn/View.php?id=CPLM010003). Here, we found that under the growth conditions used by Kosono et al. (33)*,* the acetylome of *B. subtilis* 168 the *Bs*MenE protein is acetylated at residues K86 and K471 (33). While we have no evidence that the modification of residue K86 is enzymatic, based on our data herein, it is likely that the modification of K471 observed by Kosono et al. was enzymatically introduced by *Bs*AcuA.

### *Bs*MenE acetylation leads to a growth defect

We investigated the effect of *Bs*MenE acetylation on its *in vivo* function. As mentioned before, *menE* function is essential in *B. subtilis* (34), but not in *E. coli* because this facultatively anaerobe also synthesizes ubiquinone, which is used during oxic growth. Hence, we used an *E. coli* Δ*menE* strain as a heterologous system to assess the effect of *Bs*MenE acetylation under anoxic conditions (28). To make our *in vivo* assessment of *Bs*MenE function more stringent, we used an *E. coli*Δ*menE* Δ*cobB* strain (JE24196, Table 1) to avoid deacetylating (*i.e.*, reactivating) the pool of *Bs*MenE^Ac^ generated by *Bs*AcuA. The experiment included derivatives of JE24196 that harbored either plasmid p*Bs*MENE3 (carrying *B. subtilis menE*^+^), plasmid p*Bs*AcuA (carrying *B. subtilis acuA*^+^), or both; strains carrying empty vectors (pCV3, pTAC85 Table 1) were included as controls.

**TABLE 1:** List of strains and plasmids used in this study

Strains	Genotype	Reference or source^*a*^
*B. subtilis* strains		
JE9142	*trpC2*	BGSC^*b*^
*E. coli* strains		
C41(λDE3)	*ompT hsdS* (rB^-^ mB^-^) *gal* (λDE3)	(35)
K-12 MG1655	F- lambda*- ilvG rfb-50 rph-1*	Gift from F. Blattner
Derivatives of *E. coli* K-12 MG1655		
JE24051	*menE::kan*	
JE24196	Δ*cobB*Δ*menE*	
JE24228	*menE::kan*^+^/pTAC85/pCV3	
JE24229	*menE::kan*^+^/pTAC85/pBsMENE3	
JE24230	*menE::kan*^+^/pACUA8/pCV3	
JE24231	*menE::kan*^+^/pACUA8/pBsMENE3	
JE24232	Δ*menE*Δ*cobB*/pTAC85/pCV3	
JE24233	Δ*menE*Δ*cobB*/pTAC85/pBsMENE3	
JE24224	Δ*menE*Δ*cobB*/pACUA8/pCV3	
JE24225	Δ*menE*Δ*cobB*/pACUA8/pBsMENE3	
Plasmids		
pCV1	P*_araBAD_* arabinose-inducible cloning vector, *bla*^+^	(36)
pBsMENE1	*B. subtilis menE^+^* cloned into the BspQI sites of pCV1	
pEcMENE1	*E. coli menE^+^* cloned into the BspQI sites of pCV1	
pCV3	P*_araBAD_* arabinose-inducible cloning vector, *cat*^+^	(36)
pBsMENE3	*B. subtilis menE^+^* cloned into the BspQI sites of pCV3	
pEcMENE3	*E. coli menE^+^* cloned into the BspQI sites of pCV3	
pACUA7	*B. subtilis acuA^+^* cloned into the BspQI sites of pCV3	
pTEV16	TEV protease-cleavable, N-terminal His_6_ tag overexpression vector	(36)
pBsMENE2	*B. subtilis menE^+^* cloned into the BspQI sites of pTEV16	
pBsMENE4	pBsMENE2 encoding MenE^K471A^	
pBsMENE5	pBsMENE2 encoding MenE^465-470 *E. coli*^	
pEcMENE2	*E. coli menE^+^* cloned into the BspQI sites of pTEV16	
pEcMENE4	pEcMEN2 encoding MenE^L431P^	
pEcMENE5	pEcMEN2 encoding MenE^431-436 *B. subtilis*^	

^*a*^ Unless otherwise stated, strains and plasmids were constructed during this work.

^*b*^ Bacillus Genetic Stock Center, The Ohio State University (Columbus, OH).

**Fig 8:**
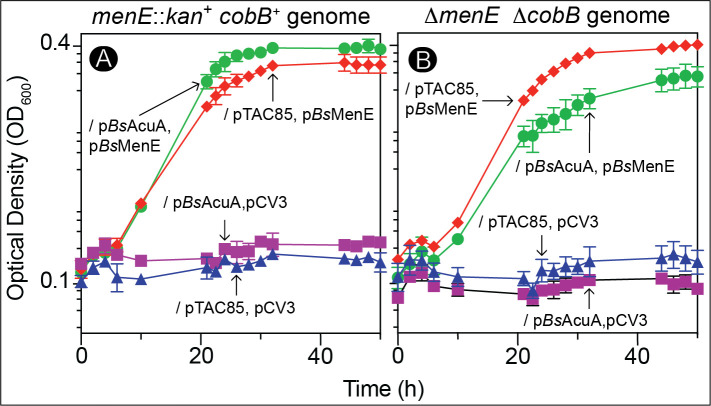
*Bs*MenE acetylation leads to a growth defect in *E. coli* lacking MenE. Panels A and B show the growth kinetics of the strains whose genotypes are described atop each panel carrying the stated plasmids. Strains JE24051 (*E. coli menE::kan^+^ cobB*^+^) and JE24196 (*E. coli* Δ*menE*Δ*cobB*) were used to construct the following strains: Panel A. JE24228 (*menE::kan^+^ cobB ^+^*/pTAC85, pCV3; triangles), JE24229 (*menE::kan^+^ cobB ^+^*/pTAC85, p*Bs*MenE, diamonds), JE24230 (*menE::kan^+^ cobB ^+^*/p*Bs*AcuA, pCV3; squares), and JE24231 (*menE cobB^+^*/p*Bs*AcuA, p*Bs*MenE; circles). Panel B. JE24224 (Δ*menE*Δ*cobB*/p*Bs*AcuA, pCV3; squares), JE24225 (Δ*menE*Δ*cobB*/p*Bs*AcuA, p*Bs*MenE; circles), JE24232 (Δ*menE*Δ*cobB*/pTAC85, pCV3; triangles), and JE24233 (Δ*menE*Δ*cobB*/pTAC85, p*Bs*MenE; diamonds). Cultures were grown in minimal medium with glycerol (22 mM) as the sole source of energy and carbon and fumarate (50 mM) as the electron acceptor under anoxic conditions that demanded menaquinone-dependent growth. pTAC85 and pCV3 are compatible cloning vectors (36, 37).

To ensure that the wild-type *Bs*MenE protein was functional, all strains were grown under anoxic conditions with glycerol as the sole source of carbon and energy and fumarate as the final electron acceptor (see Materials and Methods). Strains lacking a source of *Bs*MenE did not grow under such conditions (Fig. 8A and B; squares, triangles). In contrast, a strain carrying only plasmid pBsMENE1 (*menE^+^*) grew to full density (Fig. 8A and B; diamonds) indicating that *Bs*MenE was necessary and sufficient to support growth of the *E. coli*Δ*menE* and *E. coli*Δ*menE*Δ*cobB* strains. These results were expected since neither strain harbored the p*Bs*AcuA plasmid.

To determine the effect of *Bs*MenE acetylation on cell growth, plasmid p*Bs*ACUA8 (*acuA^+^*) was introduced into strain *menE::kan^+^ cobB ^+^*/p*Bs*MenE (Fig. 8A, circles) and strain *menE::kan^+^*Δ*cobB*/p*Bs*MenE (Fig. 8B, circles), with growth being monitored during *acuA^+^* induction. A reproducible difference in growth was measured between strains that synthesized *Bs*MenE and *Bs*AcuA (Fig. 8A and B; diamonds vs circles).

To amplify the impact of *Bs*MenE acetylation on cell growth, we repeated the experiment with *E. coli* strains lacking the *cobB* gene (encodes sirtuin deacylase, Fig. 8B). When the *B. subtilis acuA^+^* and *menE^+^* genes were induced in the *E. coli* Δ*menE*Δ*cobB*/p*Bs*MenE p*Bs*AcuA strain, the growth defect we measured was more pronounced than that of the *E. coli* Δ*menE*Δ*cobB*/p*Bs*MenE pTAC85 strain (Fig. 8B; circles vs diamonds), suggesting that acetylation of *Bs*MenE by *Bs*AcuA affected MK biosynthesis and that the absence of the CobB sirtuin deacylase exacerbated the effect of *Bs*MenE acetylation by *Bs*AcuA. We surmised that under such conditions *Bs*MenE was kept acetylated, thus inactive.

## DISCUSSION

### *Bs*MenE activity is controlled by reversible lysine acetylation (RLA)

Based on our data (Fig. 4–6 and 8), we can add the AMP-forming *Bs*MenE enzyme to the list of AMP-forming acyl-CoA synthetases whose function is controlled by RLA of a single, active site lysyl residue (9).

**Fig 9:**
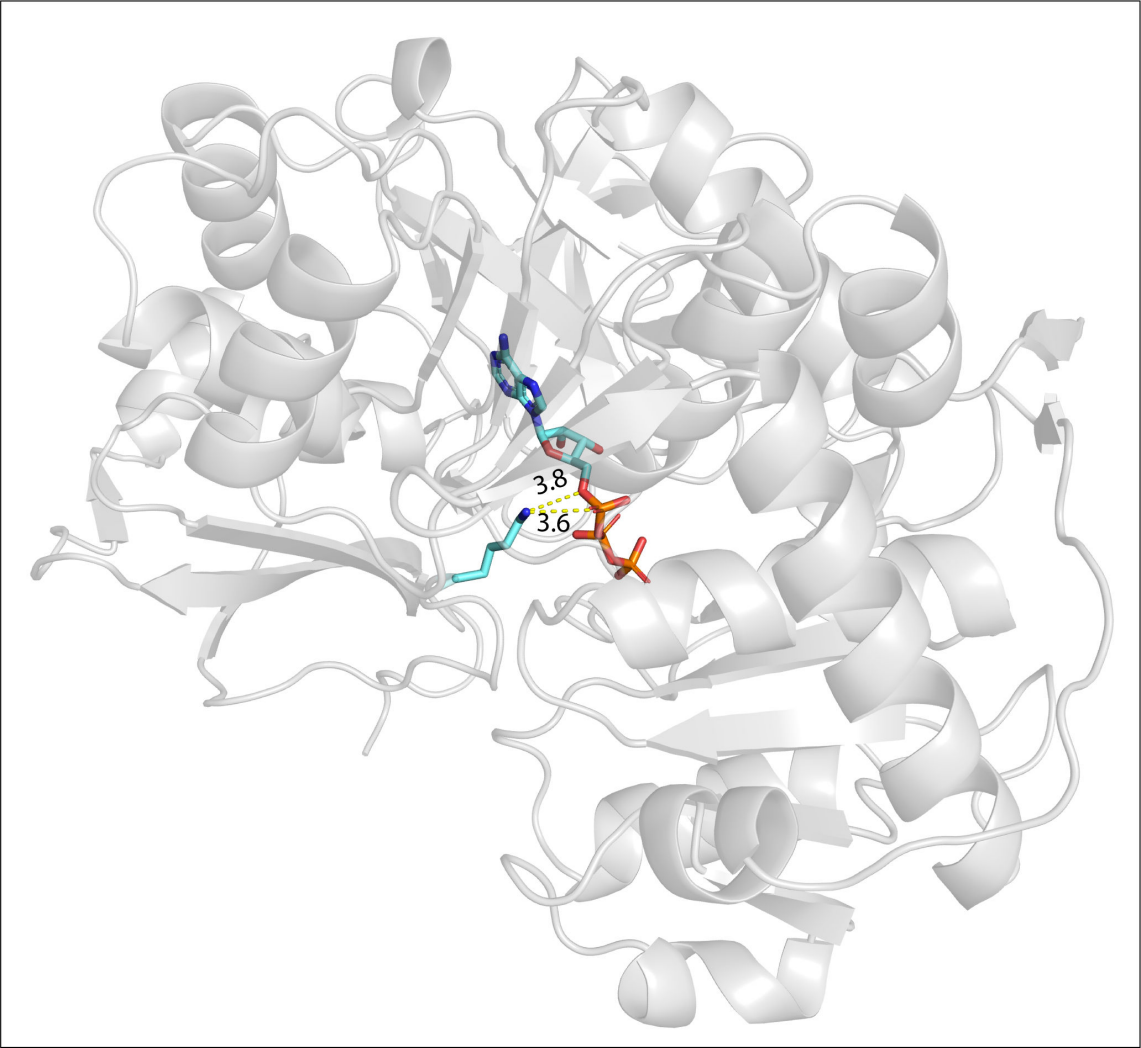
Crystal structure model of *Bs*MenE (PDB 5BUR) in complex with ATP. The distances of the interactions between the Nε amino group of residue K471 and oxygen atoms of the α phosphate group of ATP are shown in angstroms (Å). The structure is available from the RCSB Protein Data Bank (5BUR).

Figure 9 shows why residue K471 is critical to the activity of *Bs*MenE (PDB 5BUR) that yields the OSB–AMP intermediate. In the model available from the RCSB PDB, the *N*ε group of the K471 side chain interacts with two oxygens of the α phosphate moiety of ATP positioning the latter in the correct orientation for the reaction that yields the acyl-adenylate intermediate. Even though we did not assay the activity of *Bs*MenE *in vitro,* we hypothesize that acetylation of K471 would disrupt the interactions with ATP and the nucleotide would not be positioned correctly in the active site, or perhaps ATP may not even interact with the site. There is support in the literature for this hypothesis. Previous work from our laboratory showed that acetylation of active-site residues of several members of this family of proteins deactivates all the enzymes tested thus far, and that at least in the case of acetyl-CoA synthetase, acetylation specifically inhibits the formation of the acyl-adenylate without affecting the thioester bond formation reaction (9, 21, 38, 39). It is important to note that although the structure of *Bs*MenE with the OSB–AMP intermediate has been solved (PDB 5GTD), it is devoid of CoA. This point is notable because as shown by the structure of AMP-forming CoA ligases in complex with CoA, CoA binding induces a 140° rotation in the domain containing the modified lysyl residue (40–42). In the resulting conformation, these enzymes convert the acyl-adenylate to acyl-CoA releasing AMP and allowing the domain containing the active-site lysyl residue to rotate back so it can interact with ATP and initiate another catalytic cycle. However, as expected, acetyllysine cannot interact with ATP because the positive charge of the *N*ε amino group of the lysyl change has been neutralized, hence blocking the formation of the acyl-adenylate.

### Physiological consequences of *Bs*MenE acetylation

Under the conditions tested, *Bs*MenE acetylation did not cause severe phenotypes. We posit that because MK is recyclable, not catabolized, once enough MK is made and it becomes part of the electron transport chain, it will allow the cell to generate the proton motive force it needs to fuel ATP synthesis. Furthermore, RLA is used to control only a percentage of the target population, so there is always enough target activity to yield the desired product. Therefore, it is difficult to deplete MK by *Bs*MenE acetylation, especially in the strains tested, whose growth was MK dependent. Nevertheless, *Bs*MenE acetylation generated a clear, reproducible difference between strains that overproduced *Bs*AcuA and those that did not (Fig. 8A, circles vs diamonds), and we exacerbated that difference when we expressed *acuA^+^* ectopically in the absence of the chromosomal copy of the gene encoding the lysine deacetylase (CobB enzyme) (Fig. 8B, circles vs diamonds). These results were consistent with the *in vitro* data reported herein, which show that *Bs*MenE is inactivated by *Bs*AcuA and reactivated by the sirtuin deacylase. We propose that under some as-yet-undefined conditions, *B. subtilis* controls the activity of *Bs*MenE by RLA with the goal of downregulating MK biosynthesis, which would ultimately result in a lower cell energy charge, slower growth rate and cell yield.

### Why would *B. subtilis* need to control MK biosynthesis *via* RLA?

This is the question we and the reader want an answer for. Unfortunately, at present we do not have an answer, but this is the question future work will address. We speculate that in its natural habitat (soil), *B. subtilis* encounters conditions that favor the survival of slower growing cells, and that by limiting the amount of MK it synthesizes, it slows ATP synthesis and ultimately cell growth.

### Residues within the acetylation motif of *Bs*MenE are important for recognition by *Bs*AcuA of a non-acetylatable homologue

*Ec*MenE was not acetylated by a variety of known acetyltransferases, including *Bs*AcuA (Fig. 7A). However, changing the six residues upstream of the target lysine to fit more closely the consensus acetylation motif did yield acetylated *Ec*MenE protein, albeit acetylation was not as robust as *Bs*MenE (Fig. 7B, lane 3 *vs* 4), suggesting that *Bs*AcuA recognition of this area is necessary but not sufficient for strong acetylation, and that most likely changes inside and outside the acetylation motif of the *Ec*MenE protein are needed to optimize recognition by the *Bs*AcuA acyltransferase. These ideas are supported by the fact that *Bs*AcuA did not acetylate the *Bs*MenE^465-670*Ec*^ chimera, indicating that the equivalent six-residue region of *Bs*MenE (residues 431–436) is necessary for *Bs*AcuA-dependent *Bs*MenE acetylation (Fig. 7B, lane 1 vs 2).

### Why is MenE regulated in *B. subtilis* but not in *E. coli*?

**Fig 10:**
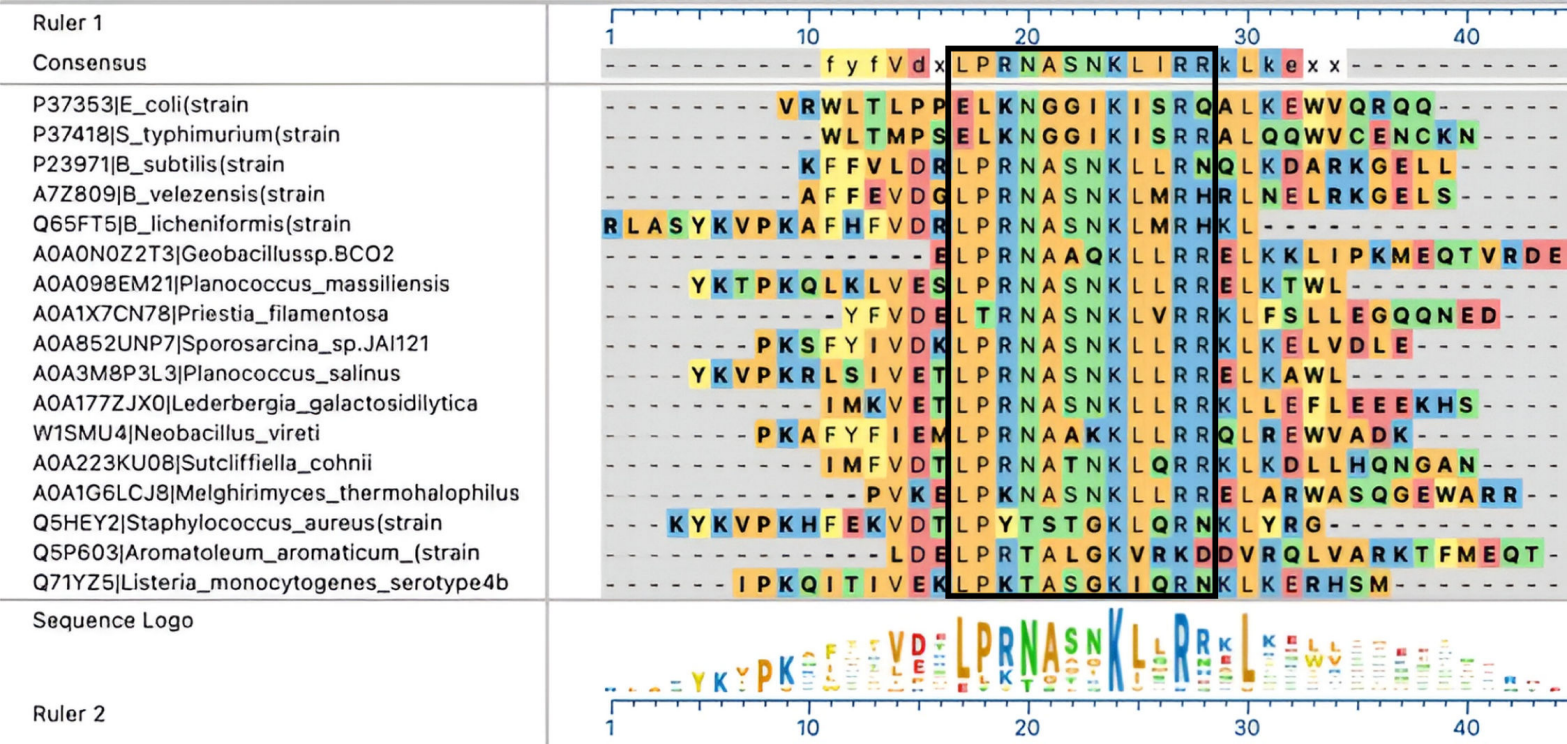
Alignment of sequences surrounding the acetylation motif at the C terminus of *Bs*MenE homologs. We generated this alignment using the online MUSCLE software program (https://www.ebi.ac.uk/Tools/msa/muscle/). This limited sample of MenE homologs was considered a starting point to get an idea of the residues that may be important for the recognition of MenE by an acetyltransferase.

This is an interesting question, which will be easier to address once we determine what prompts *B. subtilis* to regulate MK synthesis *via* MenE acetylation, and whether such a condition or signals is (are) encountered by *E. coli*. To try and get an idea of the sequence variation around the acetylation motif of MenE homologues, we aligned MenE sequences from a few bacteria using https://cplm.biocuckoo.cn/(Fig. 10) whose genomes encode a *B. subtilis menE* homolog, regardless of whether these bacteria also synthesize ubiquinone. The purpose of these comparisons was to see whether we could glean some information that would help us address the question of why MenE is regulated *via* RLA in *B. subtilis* but not in *E. coli*.

We restricted our analysis to the sequences spanning positions −7 to +4 relative to the acetylation site, using the sequence of *Bs*MenE (Fig. 10, line 3) as a point of reference. Positions −7 and −6 stood out. In all the 17 sequences we examined, those positions were occupied by Leu and Pro, respectively. Except in the case of *E. coli* and *S*. Typhimurium, where in the same positions Glu substituted for Leu, and Leu substituted for Pro. Equally striking were the changes at positions -2,–3, and −4, which changed from Ala, Ser, and Asn in *B. subtilis* to Gly, Gly, and Ile in *E. coli* and *S*. Typhimurium. All these substitutions represent substantial changes in the helicity and flexibility of the sequence, which would likely result in structural changes that may limit or completely prevent interactions between MenE from *E. coli* and *S*. Typhimurium with the *Bs*AcuA acyltransferase. These arguments may only be valid when the *Bs*AcuA is the modifying enzyme, but they do not rule out the existence of a different, as-yet-unidentified modifier that can acylate MenE from *E. coli* and *S*. Typhimurium. Of note are the changes in two other human pathogens, *i.e., Staphylococcus aureus* and *Listeria monocytogenes*, at positions -2,–3 and −4. However, MenE proteins of these bacteria were not tested in this work.

## MATERIALS AND METHODS

### Culture media, growth conditions, and chemicals

Lysogeny broth (LB, Difco) (43) was used as rich medium for *B. subtilis* and *E. coli* strains. Spizizen’s minimal medium was used to grow *B. subtilis* aerobically under defined conditions (44). Glycerol (22 mM) was used as the sole carbon and energy source in all cases. Detailed condtions used for growth analyses are described below. When added to the medium, antibiotics were present at the following concentrations: ampicillin (Fisher Scientific), 100 µg mL^−1^; erythromycin (Sigma Aldrich), 1 µg mL^−1^. 4-(2-Hydroxyethyl)-1-piperazineethanesulfonic acid (HEPES) buffer, *tris*(2-carboxyethyl)phosphine hydrochloride (TCEP), and isopropyl-β-D-1-thiogalactopyranoside (IPTG), dithiothreitol (DTT) were purchased from Gold BioTechnology; and streptothricin sulfate was purchased from G Biosciences.

### Bacterial strains

*B. subtilis* strains used in these studies were derivatives of *B. subtilis* 168. Strain JE9142 (*trpC2*) was purchased from the Bacillus Genetic Stock Center (Columbus, OH). *E. coli* strains were derivatives of *E. coli* K12 MG1655 and were constructed during this work. The *menE::kan^+^* from the KEIO collection (45) was transduced into *E. coli* wild-type and *cobB* strains using P1 phage (46). All strains used in this work are listed in Table 1.

### Plasmid constructions

All primers used in this study were synthesized by IDT (Coralville, IA) and are listed in Table 2. Genes were amplified from *B. subtilis* 168 genomic DNA or *E. coli* K12 MG1655 genomic DNA. All genes were cloned using the BspQI (New England Biolabs) cloning method (47), and cloned into complementation vectors pCV1 and pCV3 (36), in which the gene of interest is under the control of an L-(+)-arabinose-inducible promoter. For overexpression, we cloned genes into vector pTEV16 (36), in which a gene of interest is controlled by an IPTG-inducible promoter. Overexpressed genes produced proteins that were fused to a H_6_ tag at their *N*-terminus. Site-directed mutants were constructed using the QuikChange protocol (Stratagene) with pBsMENE2 or pEcMENE2 used as the template. All vectors were sequenced verified (Georgia Genomics Facility, University of Georgia, Athens, GA) and listed in Table 1.

**TABLE 2:** List of primers used in this study

Primer	Sequence
pBAD BsMenE forward	NNGCTCTTCNTTCatgCTGACAGAACAGCCCAAC
pTEV16 BsMenE forward	NNGCTCTTCNAGCatgCTGACAGAACAGCCCAAC
pBAD BsMenE reverse	NNGCTCTTCNTTAtcaTAGCAGTTCTCCTTTACGCG
BsMenE K471A forward	CAATGCGTCTAATGCGCTCTTGCGAAATC
BsMenE K471A reverse	GATTTCGCAAGAGCGCATTAGACGCATTG
BsMenE 465–470 Ec forward	CTTTGTGCTTGACCGCCTGCTCAAGAACGGCGGCATTAAGCTCTTGCGAAATCAGC
BsMenE 465–470 Ec reverse	GCTGATTTCGCAAGAGCTTAATGCCGCCGTTCTTGAGCAGGCGGTCAAGCACAAAG
pBAD EcMenE forward	NNGCTCTTCNTTCatgATCTTCTCTGACTGGCCG
pTEV16 EcMenE forward	NNGCTCTTCNAGCatgATCTTCTCTGACTGGCCG
pBAD EcMenE reverse	NNGCTCTTCNTTAttaTTGCTGACGTTGCACCC
EcMenE L431P forward	TCTGCCGCCGGAGCCAAAAAACGGCGGTAT
EcMenE L431P reverse	ATACCGCCGTTTTTTGGCTCCGGCGGCAGA
EcMenE 431–436 Bs forward	GGCTAACTCTGCCGCCGGAGCCAAGGAACGCCAGCAATAAAATTTCACGTCAGGCGC
EcMenE 431–436 Bs Reverse	GCGCCTGACGTGAAATTTTATTGCTGGCGTTCCTTGGCTCCGGCGGCAGAGTTAGCC

### Growth analyses

*E. coli* strains were grown at 37°C under anoxic conditions using Balch tubes (48). Briefly, minimal M9 medium (49) supplemented with glycerol (22 mM) and fumarate (50 mM) was brought to a boil under a stream of O_2_-free N_2_ gas. Medium (5 mL) was dispensed into Balch tubes (48), which were then capped with rubber stoppers, aluminum seals were crimped in place, and the tubes were autoclaved. Starter cultures were grown in 2 mL of rich LB medium (Tryptone 10 g/L, yeast extract 5 g/L, and sodium chloride 5 g/L) in borosilicate glass tubes (13 × 100 mm) for 14–16 h at 37°C in an *innova43* (New Brunswick Scientific) gyratory incubator shaking at 180 rpm. Balch tubes were inoculated with 1.5% (v/v) of an overnight starter culture using a sterile syringe with a 22G needle previously flushed with O_2_-free N_2_ gas. Prior to inoculation, ethyl alcohol was applied to the top of the stoppers of Balch tubes and flamed to sterilize their surface. Cells were grown without shaking, and cell density was monitored at 600 nm (Spectronic 20D) as a function of time. Each strain was grown under identical conditions in duplicate. The data were analyzed using the GraphPad Prism v6 software package (GraphPad Software).

### Purification of H_6_-tagged proteins

All proteins were overproduced in *E. coli* strain C41(λDE3) cells (35) carrying the appropriate pTEV16 vector (Table 1). Cells were grown in 1.5 L of Terrific Broth (Cold Spring Harbor Laboratory Protocols) at 37°C to an OD_600_ of 0.3–0.4. At this cell density, IPTG was added to a final concentration of 500 µM, and cultures were incubated overnight at 15°C in a 2.8 L flask in an *innova44* (New Brunswick Scientific) gyratory shaker at 160 rpm. Cells were harvested by centrifugation at 6,000 × *g* for 15 min, and the cell paste was frozen at −80°C until use.

Protein was purified as described elsewhere (50). Briefly, cell paste was resuspended in HEPES buffer (50 mM, pH 7.5 at 4°C) containing NaCl (500 mM) and imidazole (20 mM), 1 µg mL^−1^ lysozyme, 25 µg mL^−1^ DNase, and 0.5 mM phenylmethanoesulfonyl fluoride (PMSF, Fisher Scientific). Cells were broken by sonication, and cellular debris was removed by centrifugation. The clarified cell-free extract was loaded onto a 5 mL Ni-NTA affinity column (HisPur; ThermoFisher Scientific) The column was washed with 10 bed volumes of bind buffer (HEPES, 50 mM, pH 7.5) containing NaCl (500 mM) and imidazole (20 mM), followed by six bed volumes of wash buffer (HEPES 50 mM, pH 7.5) containing NaCl (500 mM) and imidazole (40 mM). Protein was eluted off the column with six bed volumes of elution buffer (HEPES 50 mM, pH 7.5) containing NaCl (500 mM) and high imidazole (500 mM). Fractions of eluted protein were pooled and dialyzed against HEPES buffer (50 mM, pH 7.5 at 4°C) with decreasing amounts of NaCl down to 150 mM. Purified protein was flash frozen in liquid nitrogen and stored at −80°C until use.

### *In vitro* acetylation assay

Reaction mixtures (25 µL) containing HEPES buffer (50 mM, pH 7 at 24°C), TCEP (1 mM), [1-^14^C]Ac-CoA (20 µM; spec. rad. = 56.8 mCi/mmol), acetyltransferase (1 µM), and purified H_6_-MenE proteins (5 µM) were incubated at 37°C for 1 h. Reaction mixtures lacking enzyme or target protein were used as controls. Reactions were quenched by the addition of SDS loading buffer. Samples from reaction mixtures were resolved by SDS-PAGE, and radioactivity distribution was visualized using a phosphor imager after 16 h of exposure. A Typhoon Trio+Variable Mode Imager (GE Health Life Sciences) and ImageQuant v5.2 software were used to image the phosphor screen.

### Protein deacetylation assay

Various H_6_-MenE proteins were acetylated with [1-^14^C]Ac-CoA as stated above. Excess [1-^14^C]Ac-CoA was removed by buffer exchange, and *Bs*MenE^Ac^ was incubated with *Bs*SrtN (3 µM final concentration), *Bs*SrtN and NAD^+^ (1 mM), *Bs*SrtN with NAD^+^ (1 mM), and nicotinamide (1 mM), or *Bs*AcuC (3 µM) in HEPES buffer (50 mM [pH 7]) for 2 h at 37°C. Samples were resolved by SDS-PAGE, and deacetylation was monitored phosphor imager as described above.

### Acetylation motif weblogo

Acetylation motif was determined by aligning the amino acid sequences of acetylatable acid:CoA ligase. Sequences aligned were as follows: *E. coli* Acs, *S*. Typhimurium Acs, *S*. Typhimurium PrpE, *B. subtilis* AcsA, *R. palustris* Acs, *R. palustris* HbaA, *R. palustris* IbuA (RPA2302), *R. palustris* PrpE (RPA4504), *R. palustris* FadD (RPA4267), *R. palustris* BadA (RPA0661), *R. palustris* AliA (RPA0651), *R. palustris* LcsA (RPA4421), *R. palustris* FcsA (RPA1702), *R. palustris* HcsA (RPA1003), and *Micromonospora aurantiaca* Acs (Micau_0428). The sequence of residues around the acetylated lysine were put into a web-based software (http://weblogo.berkeley.edu/logo.cgi) and used to create the weblogo.

### LC/MS/MS peptide fingerprinting analysis

Purified MenE was resolved using a 12% SDS-PAGE gel that was stained with Coomassie Blue G-250 to visualize proteins (51). The LC/MS/MS was performed using a Thermo-Fisher LTQ Orbitrap Elite Mass Spectrometer coupled with a Proxeon Easy NanoLC system (Waltham, MA) located at the Proteomics and Mass Spectrometry Facility of the University of Georgia. The enzymatic peptides were loaded into a reverse-phase column (self-packed column/emitter, 0.1× ~ 150 mm ID, with 200 Å 5 µM Bruker MagicAQ C18 resin), then directly eluted into the mass spectrometer at the flow rate of 450 nL/min. Briefly, the two-buffer gradient elution (0.1% formic acid as buffer A and 99.9% acetonitrile with 0.0.1% formic acid as buffer B) starts with 0% B, holds at 0%B for 2 min, then increases to 12% B in 25 min, to 30% B in 25 min, to 50% B in 10 min, and to 95% B in 10 min. The data-dependent acquisition (DDA) method was used to acquire MS data. A list of expected precursor ions was generated for monitoring possible N-terminal peptides with one missed cleavage. A survey MS scan was acquired first (*m/z* 350–1500), and then the top four ions in the precursor list were selected for CID followed by HCD MS/MS analysis with an isolation width of 2 *m/z*. If the no peptide ion in the precursor list was found, the most abundant ions were chosen for MS/MS analysis. Both MS and MS/MS scans were acquired by Orbitrap at resolutions of 120,000 and 15,000, respectively. Data were acquired using Xcalibur software (version 2.2, Thermo Fisher Scientific). Protein identification and modification characterization were performed using Thermo Proteome Discoverer (version 1.4) with Mascot (Matrix Science, London, UK) and Uniprot *Bacillus subtilis* database. The search parameters include i) precursor mass tolerance: 10 ppm, ii) fragment mass tolerance: 0.02 Da, iii) modification: oxidation of methionine, iv) validated with percolator (decoy database), targeted FDR (restrict/relax): 0.01/0.05.

## Data Availability

All the data generated by this work are contained in this paper.
